# Integrated Single-cell and Transcriptome Sequencing Analyses Identified PREX1 as an Immune-related Prognostic Biomarker for Liver Hepatocellular Carcinoma

**DOI:** 10.7150/ijms.94812

**Published:** 2024-06-03

**Authors:** Jing Yang, Lin-Yin Fan, Kai-Yuan Shi

**Affiliations:** 1 Department of Diagnostic Ultrasound Imaging & Interventional Therapy, Zhejiang Cancer Hospital, Hangzhou Institute of Medicine (HIM), Chinese Academy of Sciences, Hangzhou, 310022, China.; 2 Department of Radiology, Zhejiang Cancer Hospital, Hangzhou Institute of Medicine (HIM), Chinese Academy of Sciences, Hangzhou, Zhejiang, 310022, China.

**Keywords:** PREX1, Liver hepatocellular carcinoma, Prognosis, Immune infiltration, Single cell analysis

## Abstract

**Background:** PtdIns (3,4,5) P3-dependent Rac exchanger 1 (PREX1), also known as PREX1, a member of the Rac guanine nucleotide exchange factors (Rac-GEF) family. Studies have suggested that PREX1 plays a role in mediating oncogenic pathway activation and controlling various biological mechanisms in different types of cancer, including liver hepatocellular carcinoma (LIHC). However, the function of PREX1 in the pathogenesis of LIHC and its potential role on immunological regulation is not clearly elucidated.

**Methods:** The expression level and the clinical role of PREX1 in LIHC was analyzed based on database from the Cancer Genome Atlas (TCGA), TNM plotter and University of Alabama Cancer Database (UALCAN). We investigated the relationship between PREX1 and immunity in LIHC by TISIDB, CIBERSORT and single cell analysis. Immunotherapy responses were assessed by the immunophenoscores (IPS). Moreover, biological functional assays were performed to further investigate the roles of PREX1 in liver cancer cell lines.

**Results:** Higher expression of PREX1 in LIHC tissues than in normal liver tissues was found based on public datasets. Further analysis revealed that PREX1 was associated with worse clinical characteristics and dismal prognosis. Pathway enrichment analysis indicated that PREX1 participated in immune-related pathways. Through CIBERSORT and single cell analysis, we found a remarkable correlation between the expression of PREX1 and various immune cells, especially macrophages. In addition, high PREX1 expression was found to be associated with a stronger response to immunotherapy. Furthermore, *in vitro* assays indicated that depletion of PREX1 can suppress invasion and proliferation of LIHC cells.

**Conclusion:** Elevated expression of PREX1 indicates poor prognosis, influences immune modulation and predicts sensitivity of immunosuppression therapy in LIHC. Our results suggested that PREX1 may be a prognostic biomarker and therapeutic target, offering new treatment options for LIHC.

## Introduction

Liver Hepatocellular carcinoma (LIHC) is the main type of hepatic tumor and is a leading cause of cancer-related deaths worldwide 1, 2. The early stages of LIHC are asymptomatic, making it difficult to diagnose until it reaches an advanced stage 3. Despite great advances in surgical procedures, liver transplantation and the targeted agent treatment, incidence and mortality of LIHC have been increasing. Therefore, gaining insights into reliable biomarkers to discriminate LIHC and molecular mechanisms to target LIHC will be extremely instrumental for patients.

PtdIns(3,4,5)P3-dependent Rac exchanger (PREX1) is part of the Rac guanine nucleotide exchange factors (Rac-GEF) family, which includes three members: PREX1, PREX2 (PREX2a), and PREX2b 4, 5. PREX1 restrains the activity of phosphatase and tensin congeners (PTEN) and consequently regulates downstream PI3K pathway. By facilitating exchange of GDP for GTP, subsequent activation of Rac is involved in diverse biological process, such as cellular migration, adhesion, actin cytoskeletal rearrangement and the release of reactive oxygen species (ROS) 6, 7. Recently, a wealth of studies focused on the function of PREX1 in tumors, such as prostate cancer 8, melanoma 9, and breast cancer 10, which contributed to enhanced aggressiveness and poor prognosis. However, the role of PREX1 in the pathogenesis of LIHC is not clearly illuminated.

The tumor microenvironment, which includes the extracellular matrix, stromal cells, innate and adaptive immune cells, plays a vital role in LIHC progression. LIHC remains a typically inflammatory tumor and holds special immune signatures in the context of viral hepatitis and cirrhosis. LIHC is characterized by a distinct desmoplastic stroma, generally occurs accompanied by the infiltration of immune and inflammatory cells 11, 12. Immune cells infiltration has been shown to be a predictor of overall survival (OS) and disease-free survival (DFS) in LIHC 13. Intriguingly, PREX1 has been found to modulate the function of neutrophils and macrophages 14, 15, but its relationship with immunity in LIHC remains unclear.

In this research, we found high expression of PREX1 promoted LIHC progression through bioinformatics analysis and cell function assays, providing a prognostic indicator. Furthermore, our findings revealed that PREX1 may shape the immune microenvironment of liver cancer and enhance sensitivity to immunosuppressive treatment. These results have important implications for the development of novel therapeutic strategy for combating LIHC.

## Material and methods

### Data acquisition and reanalysis

Tumor Immune Estimation Resource (TIMER) 16, which consists of differential gene expression between tumor samples and normal tissues, was used to explore mRNA expression of PREX1 in various cancer. The RNA sequencing data was retrieved from The Cancer Genome Atlas (TCGA) database, and subsequently analyzed with the “limma” R package. Meanwhile, TNM plotter 17 was used to analyze the mRNA expression of PREX1 in LIHC tissues. University of Alabama Cancer Database (UALCAN) 18 was utilized to analyze the protein level of PREX1 in LIHC.

### Survival analysis

According to the median expression level of PREX1, TCGA-LIHC samples were separated into two groups of high and low expression. The survival analysis was performed to determine whether the expression of PREX1 was related to the prognosis in LIHC patients.

### Relationship between PREX1 and clinical information

Univariate and multivariate Cox regression analysis was conducted to screen independent prognostic factors. By “ggplot2” and “ComplexHeatmap” packages, we evaluated the relationships between PREX1 and multiple clinical factors.

### Differentially expressed gene analysis

We separated TCGA-LIHC samples into two groups of high and low PREX1 expression groups according to the median value of PREX1. For screening differentially expressed genes (DEGs), we used the “limma” R package and set |log2-fold change (FC)| > 1.5 and p values of < 0.05 as the thresholds for DEGs. In this study, we also conduct co-expression analysis to evaluate the relationships of PREX1 with other genes by analyzing TCGA-LIHC samples.

### Functional enrichment analysis

By using the “limma” R package, we screened differentially expressed genes (DEGs) between the above two groups. Next, functional enrichment analyses was conducted on the DEGs by applying the “clusterProfiler” R package, including GO pathway and KEGG pathway analyses. Furthermore, we conducted gene set enrichment analysis (GSEA) by utilizing the R packages “clusterProfiler” and “limma”.

### Correlation analysis of PREX1 and its related genes with immunity in LIHC

TIMER was used for analyzing the correlation of PREX1 and its related genes with several immune cell types. And CIBERSORT 19 were utilized to analyze the relationship between PREX1 expression level and the degree of infiltrating immune cells. Moreover, the relationship of PREX1 with immune signatures was investigated, such as immunostimulators, immunoinhibitors, MHC molecules, chemokines and its receptors in LIHC by TISIDB database 20. Besides, the Wilcoxon test and the ESTIMATH algorithm were conducted to analyze the differences in TME scores between two sets of high and low PREX1 expression, including ESTIMATE score, immune score and stromal score.

### Expression of PREX1 and drug sensitivity analysis in LIHC

Through analysis of Cancer Immunome (TCIA) database 21, we performed immunophenoscore (IPS) analyses for both PD1 and CTLA4 inhibitors to investigate the predictive value of PREX1 in terms of the sensitivity to immunotherapy in LIHC patients. IPS between two groups of high and low PREX1 expression was analyzed to compare the efficacy of immune checkpoint inhibitor therapy by using Kruskal-Wallis test. We analyzed the half-maximal inhibitory concentration (IC50) of specific inhibitors by the "oncoPredict" R package. IC50 between two groups of high and low PREX1 expression was analyzed to compare the efficacy of chemotherapy by using Kruskal-Wallis test.

### Single cell analysis

We downloaded the 10x Genomics sequencing expression raw matrix from GEO database (GSE166635 22), including 2 LIHC samples. Seurat R (version 4.3.0) package was applied to analyze raw data with R studio. Cells were included according to following criteria. (1) less than 20% mitochondria-related genes expressed. (2) more than 200 but less than 6,500 genes expressed. 18710 cells were obtained for analysis after normalization. We then applied Harmony R package for batch integration 23, 24. Then, the tSNE and UMAP analysis were conducted for reduction and visualization of genes. The signatures from cell marker database and previous publications were used for cell detailed annotation 25.

We also explored the expression of PREX1 in different types of immune cells by conducting single cell analysis based on datasets from Tumor Immune Single-Cell Hub (TISCH) website 26. Retrieval condition: Gene: PREX1; cell type annotation: major lineage; all lineage. The expression of PREX1 in 16 cell clusters is showed in a heatmap. Moreover, the PREX1 expression level in each cell cluster in the LIHC _GSE140228_Smartseq2 datasets was disclosed.

### Cells and reagents

Human Hepatocellular Carcinoma cells Huh7 and SMMC7721 were purchased from Chinese Academy, Shanghai. Huh7 and SMMC7721 were cultured in Dulbecco's modified Eagle's medium (DMEM) (Hyclone) containing 10% FBS (Gibco, USA). Small interfering RNAs (siRNAs) were obtained by GenePharma (Shanghai, China). LipofectamineTM 2000 was applied for transfection with the manufacturer's protocol. The siRNA sequences of PREX1 were as follow: 5′-GAAGUAACAGCUCCUACUUdTdT-3′; anti-sense 5′-AAGUAGGAGCUGUUACUUCdTdT-3′.

### Western blot

Whole cell lysis was produced by using RIPA buffer with protease inhibitor. Protein samples were separated by sodium dodecyl sulfate polyacrylamide gel electrophoresis (SDS-PAGE) and transferred onto Polyvinylidene fluoride (PVDF) membranes. After blocking with 5% non-fat milk in TBS-T, membranes were incubated with the primary antibody and secondary antibody. Protein bands were detected by using Image Acquisition using ImageQuant™ LAS 4000 (GE Healthcare Life Sciences).

### RNA extraction and reverse transcription PCR

RNA of cell lines was isolated using Trizol reagent (Invitrogen) and then reversely transcripted into cDNA. Real-time PCR was performed using SYBR Green PCR Master Mix (DBI Bioscience) and ABI PRISM 7900 Sequence Detection System (Applied Biosystems). Results were normalized to GAPDH for mRNA measurement. Fold change was calculated by the 2-ΔΔCt method where ΔΔCt=ΔCt (Target) - ΔCt (Reference). The forward and reverse primer sequences were used as follows: PREX1: 5′-GGCATTCCTGCATCGCATC-3′ and 5′-CGGGTGTAAACAATACTCCAAGG -3′.

### CCK8 assay

Cells transfected with negative control siRNA (si-NC) or si-PREX1 were seeded in 96-well plate. Each well was added with 10 μl CCK8 solution on each day of the subsequent days. After 2h of continuous incubation, the absorbance at 450 nm of each well was assessed and the cell proliferation rate was calculated.

### Transwell invasion assays

Transwell assays were conducted for exploring the invasive ability of cells transfected with si-NC or si-PREX1. A total of 5×10^4^ cells with Transwell BD Matrigel (Corning, USA) were seeded in the upper chamber and serum-starved overnight. Meanwhile, the lower chamber was added with culture medium containing 20% FBS. After 48h culturing, invading cells were fixed in methanol and then stained with crystal violet. The invasive cells were shot and calculated.

### Statistical analysis

The main analysis for PREX1 expression was completed by using online databases as stated. The Kaplan-Meier method was applied for survival outcomes and log-rank tests were conducted. Correlation analysis was performed using Spearman's correlation. For the above studies, the results are considered significant when *p<0.05, **p < 0.01, or ***p< 0.001.

## Results

### High expression of PREX1 in LIHC demonstrates poor outcome

**Figure 1:**
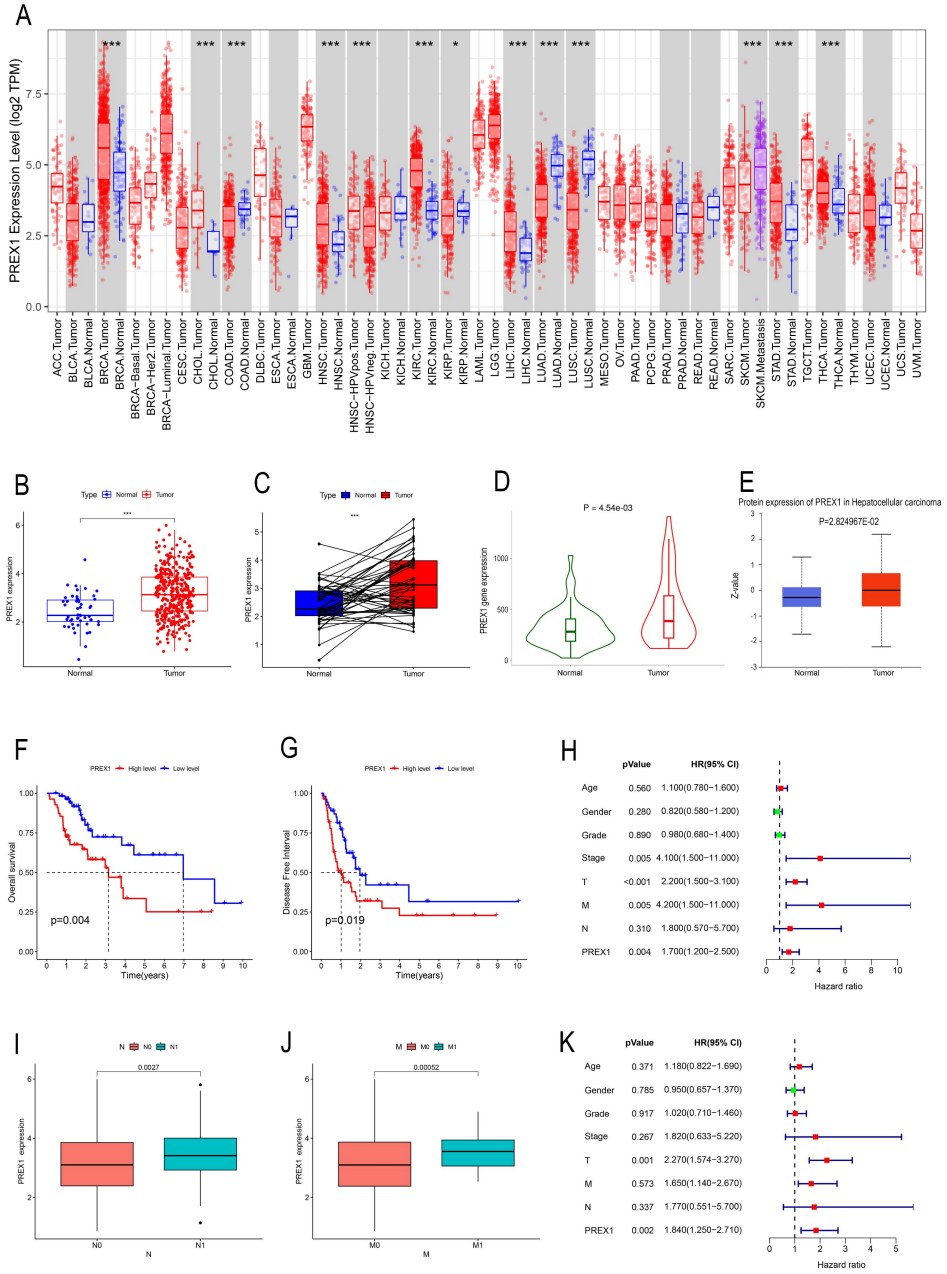
The clinical role of PREX1 in LIHC. (A) Expression of PREX1 in various cancers analyzed by TIMER. (B) The difference analysis of PREX1 expression in normal and tumor TCGA-LIHC samples. (C) Pairwise difference analysis of PREX1 in normal and tumor TCGA- LIHC samples. (D) Expression of PREX1 analyzed by TNM plotter. (E) The protein level of PREX1 analyzed by UALCAN database. (F) OS and (G) DFS in high and low PREX1 group in TCGA-LIHC cohort. (I-J) Clinical correlation of PREX1. (H, K) Univariate and multivariate analysis of PREX1.

TIMER was employed to investigate the expression of PREX1 between tumor and normal tissues in multiple cancers. Our results demonstrated that mRNA expression of PREX1 was significantly higher in several malignant tumors than in normal tissues, including LIHC (Figure 1A). Then we measured the mRNA expression of PREX1 in LIHC by using TCGA datasets and TNM plotter. Our analysis confirmed a remarkable increase of PREX1 in liver tumor tissues relative to normal liver tissues (Figure 1B-D). Moreover, the protein expression of PREX1 was proved to be higher in liver tumor samples than normal liver tissues (Figure 1E). Kaplan-Meier analysis demonstrated that patients with high expression of PREX1 showed poor prognosis, including OS and DFS (Figure 1F-G). Then, the clinical correlation of PREX1 was analyzed in LIHC patients. The heatmap revealed the distribution of clinical information for two different expression groups of PREX1(Supplementary Figure 1). A remarkably higher level of PREX1 was found in patients with worse M- and N-stage, disclosing its promoting role on tumor metastasis (Figure 1I-J). Univariate and multivariate analyses indicated that PREX1 was an independent risk factor for LIHC (Figure 1H, K).

### GO, KEGG, and GSEA identify PREX1-related signaling pathways in LIHC

**Figure 2:**
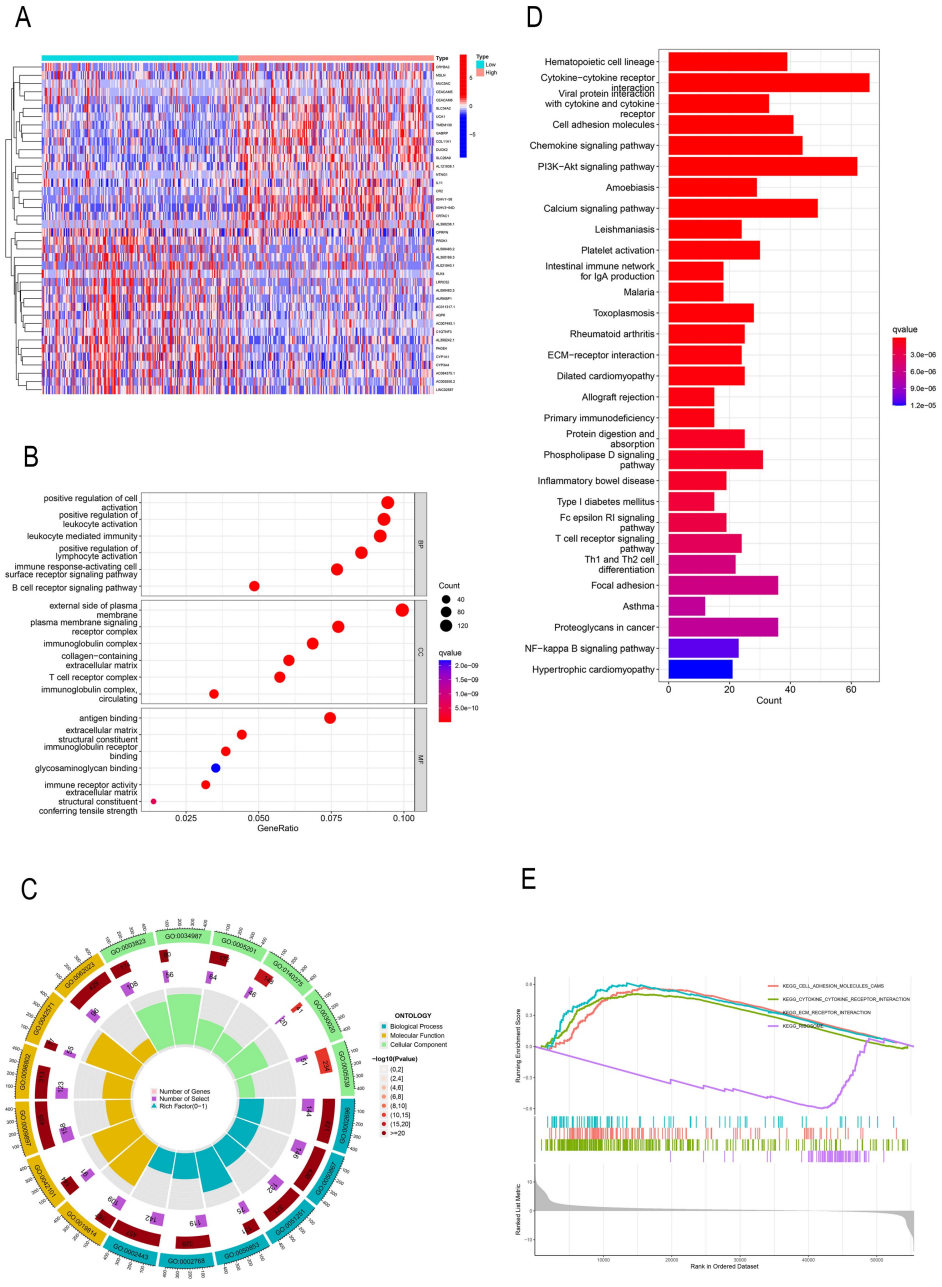
PREX1-related genes were enriched in various immune-related pathways. (A) The top 40 correlated genes in the high and low PREX1 expression groups. (B, C) GO pathway enrichment analysis in LIHC. (D) KEGG pathway enrichment analysis in LIHC. (E) GSEA enrichment analysis in LIHC.

In order to investigate the effect of PREX1 on LIHC development, a total of 2154 PREX1 significantly related genes were screened. The top 40 genes bearing positive and negative correlation with PREX1 were shown in the heat map (Figure 2A). Next, the significantly upregulated genes were applied to conduct GO, KEGG, and GSEA analyses. GO analysis manifested that PREX1 mainly participated in biological processes such as leukocyte mediated immunity, lymphocyte activation and immune response-activating cell surface receptor signaling pathway (Figure 2B, C; Supplementary Table 1). KEGG pathway analysis displayed cytokine-cytokine receptor interaction, PI3K-Akt signaling pathway, chemokine signaling pathway, cell adhesion molecules, etc. (Figure 2D). ECM receptor interaction, adhesion molecules and cytokine-cytokine receptor interaction were all enriched in GSEA analyses (Figure 2E).

### PREX1 and its related genes are associated with infiltration of immune cells in LIHC

**Figure 3:**
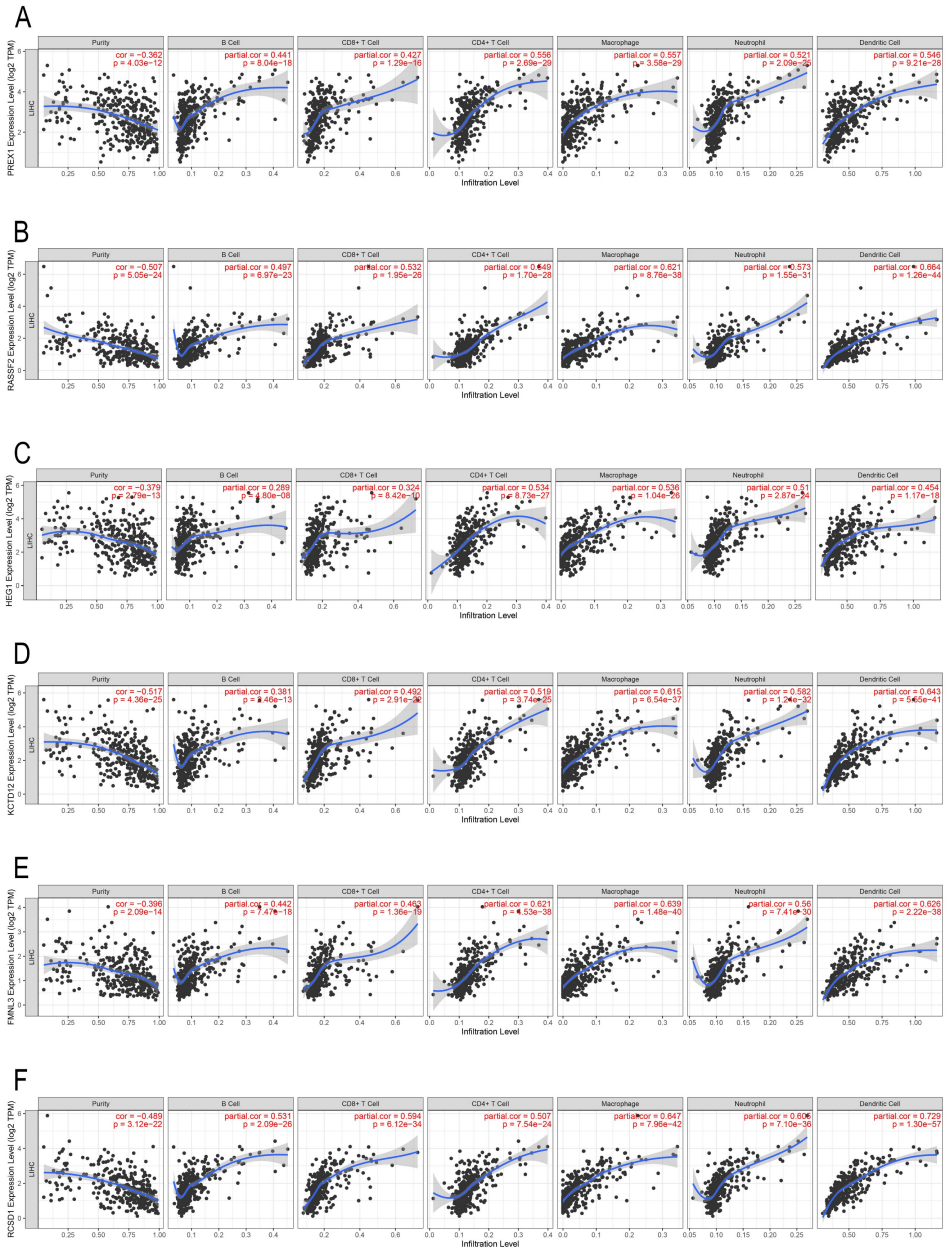
The correlation of PREX1 and its related genes with the level of immune infiltration in LIHC. Correlation of PREX1 (A), RASSF2 (B), HEG1 (C), KCTD12 (D), FMNL3 (E) and RCSD1(F).

In the past decade, mounting research revealed that tumor infiltrating lymphocytes (TILs) have general influence on tumor development 27, 28. We employed the TIMER database to assess the correlation between PREX1 expression and different TILs in LIHC. PREX1 was correlated with all six immune cells, particularly with CD4^+^ T cells and macrophages (Figure 3A). Additionally, the correlation between genes significantly related to PREX1 (RASSF2, HEG1, KCTD12, FMNL3 and RCSD1; Supplementary Figure 2) and TILs were investigated, and these genes were all confirmed to be significantly correlated with immune cells (Figure 3B-F).

**Figure 4:**
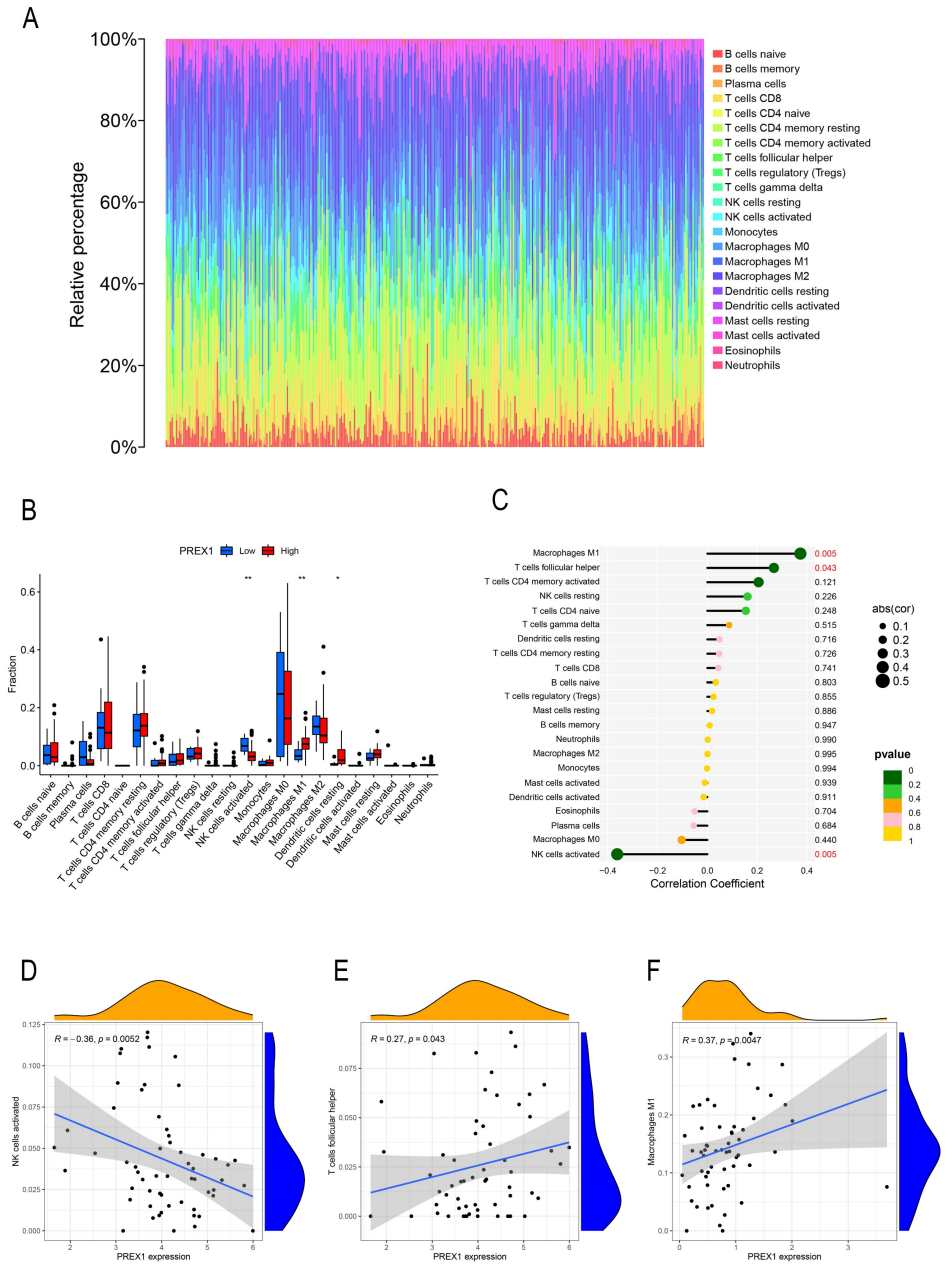
The relationship between PREX1 expression and 22 immune cells in LIHC. (A) The proportion of 22 immune cells in each LIHC sample was determined by CIBERSORT. (B) The difference of 22 immune cells proportion in the PREX1 high level group compared to the PREX1 low level group. (C) The correlation between PREX1 expression and immune cells. (D-F) Scatter plots depicting the correlations between PREX1 and specific immune cells.

By the analysis of CIBERSOTE, we investigated the proportion of 22 immune cell types in TCGA-LIHC patients (Figure 4A). To analyze whether PREX1 expression level differed in the aspect of 22 immune cells in LIHC, samples were divided into low and high PREX1 expression groups. NK cells activated, M1 macrophages and Dendritic cells resting was significantly different in the above groups (Figure 4B). We then analyzed the correlation of immune cells with PREX1 in LIHC by using the R software. T cell follicular helper and M1 macrophages was positively correlated with PREX1, while NK cells activated was negatively correlated with PREX1 (Figure 4C). To further explore the statistical significance of these findings, we utilized scatter plots to show the correlations between PREX1 expression and specific immune cell (Figure 4D-F).

### PREX1 is significantly related with multiple immune factors in LIHC

**Figure 5:**
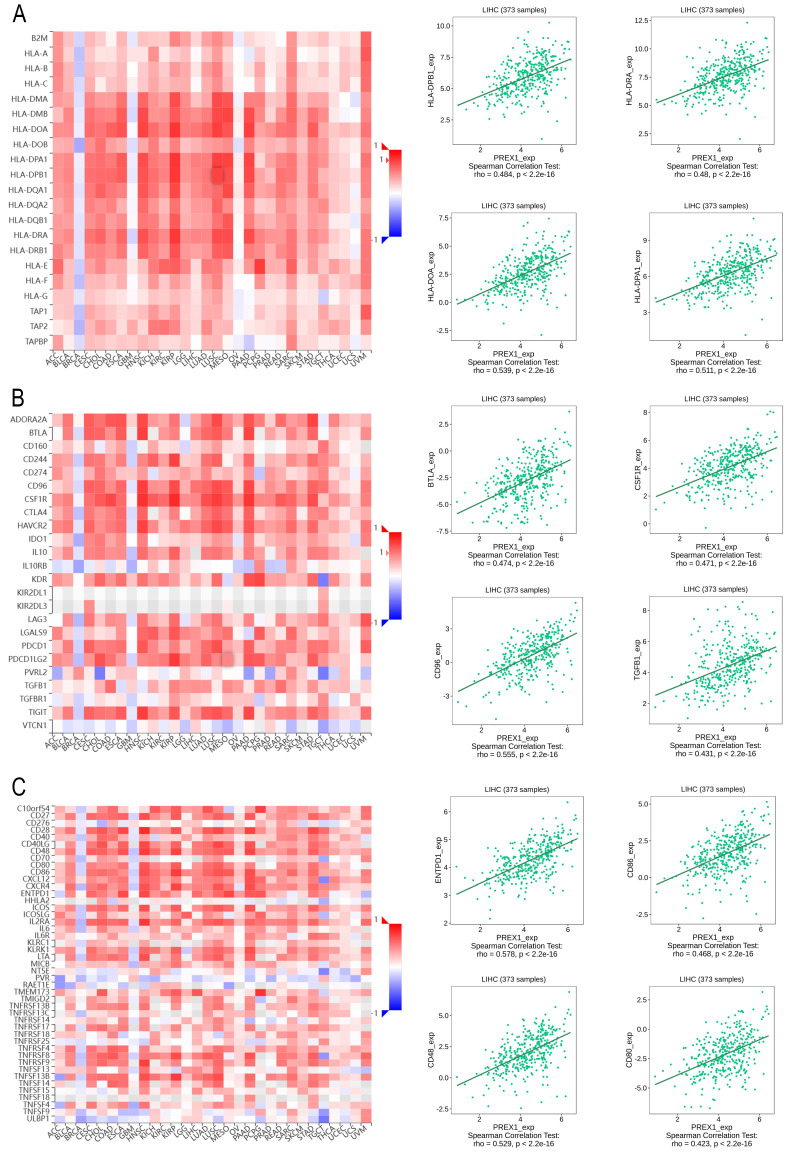
Correlation of PREX1 expression with MHC molecules, immune inhibitors and immune stimulators in LIHC by analyzing TISIDB database. (A) Correlations between PREX1 and MHC molecules (plus the four MHC molecules with the highest correlation). (B-C) Correlations between PREX1 and multiple immunomodulators (plus the four immune inhibitors and immune stimulators with the highest correlation, respectively).

**Figure 6:**
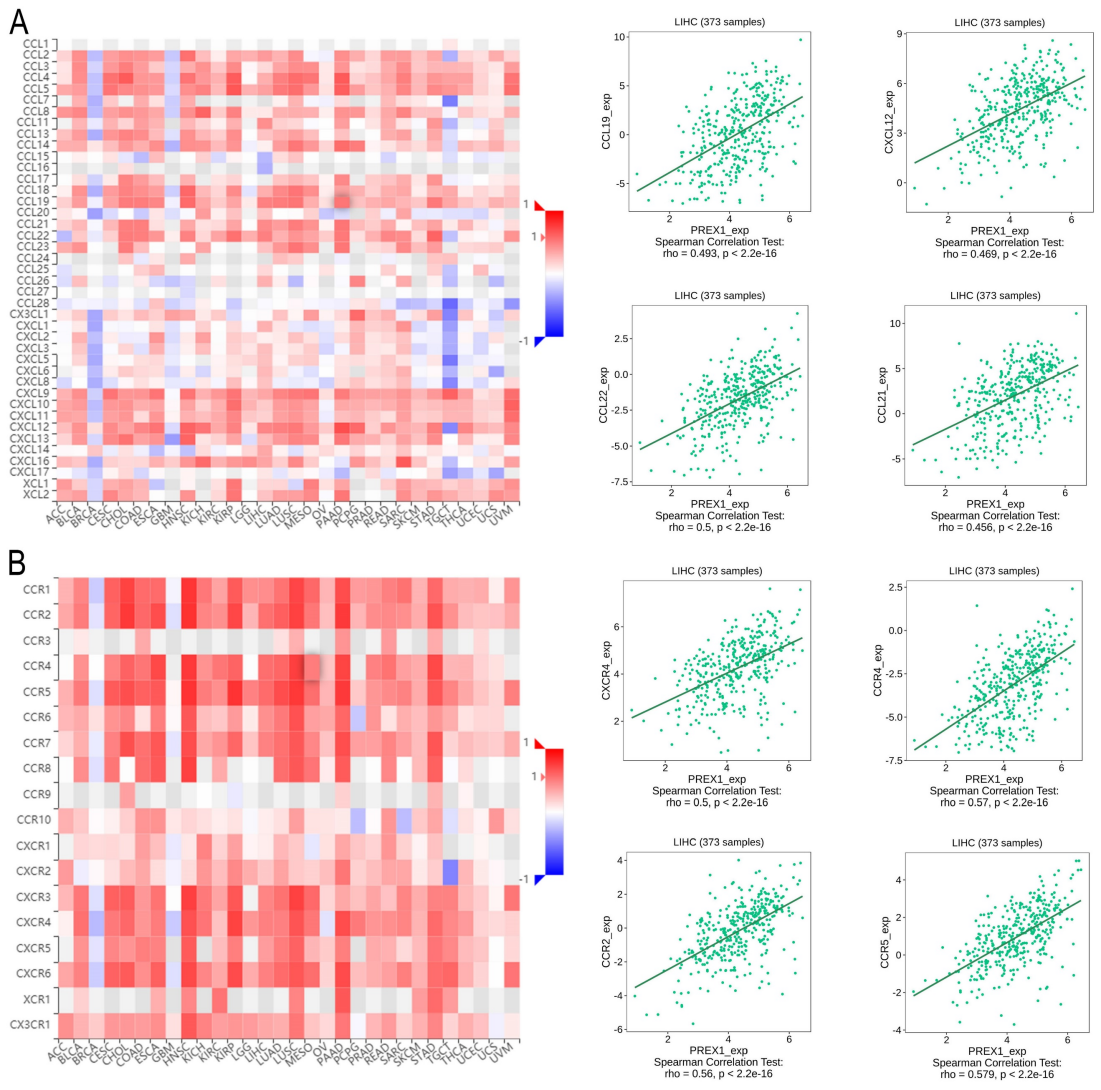
Correlation of PREX1 expression with chemokines and its' receptors in LIHC by analyzing TISIDB database. (A-B) Correlations between PREX1 and various chemokines (or receptors) [plus the four chemokines (or receptors) with the highest correlation, respectively].

Next, we explored the association between PREX1 expression and various immune signatures to broaden the understanding of the relationship between PREX1 and TILs. The MHC molecules, such as HLA-DPA1, HLA-DOA, HLA-DRA, and HLA-DPB1, showed significant correlation with PREX1 based on the analysis of TISIDB database (Figure 5A). PREX1 also regulated various immune inhibitors and stimulators, such as CSF1R, CD96, BTLA and TGFB1 (immune inhibitor, Figure 5B), as well as CD86, CD80, ENTPD1, and CD48 (immune stimulators, Figure 5C). Moreover, we investigated the chemokines and its receptors that may be modulated by PREX1. It can be seen that the chemokine highly related to PREX1 are the CXCL12, CCL19, CCL21, CCL22 (Figure 6A), and the receptors highly related to PREX1 are the CXCR4, CCR2, CCR5, CCR4 (Figure 6B).

Finally, we performed the ESTIMATH algorithm and the Wilcoxon test to evaluate the differences of PREX1 expression in TME scores, including immune score, stromal score, and ESTIMATE score. The group with high PREX1 level revealed a higher immune score, stromal score, and ESTIMATE score (Supplementary Figure 3). As a result, PREX1 was demonstrated to widely regulate many different kinds of immune molecules, thereby influencing immune infiltration in LIHC microenvironment.

### PREX1 is highly expressed in macrophage using single cell analysis

**Figure 7:**
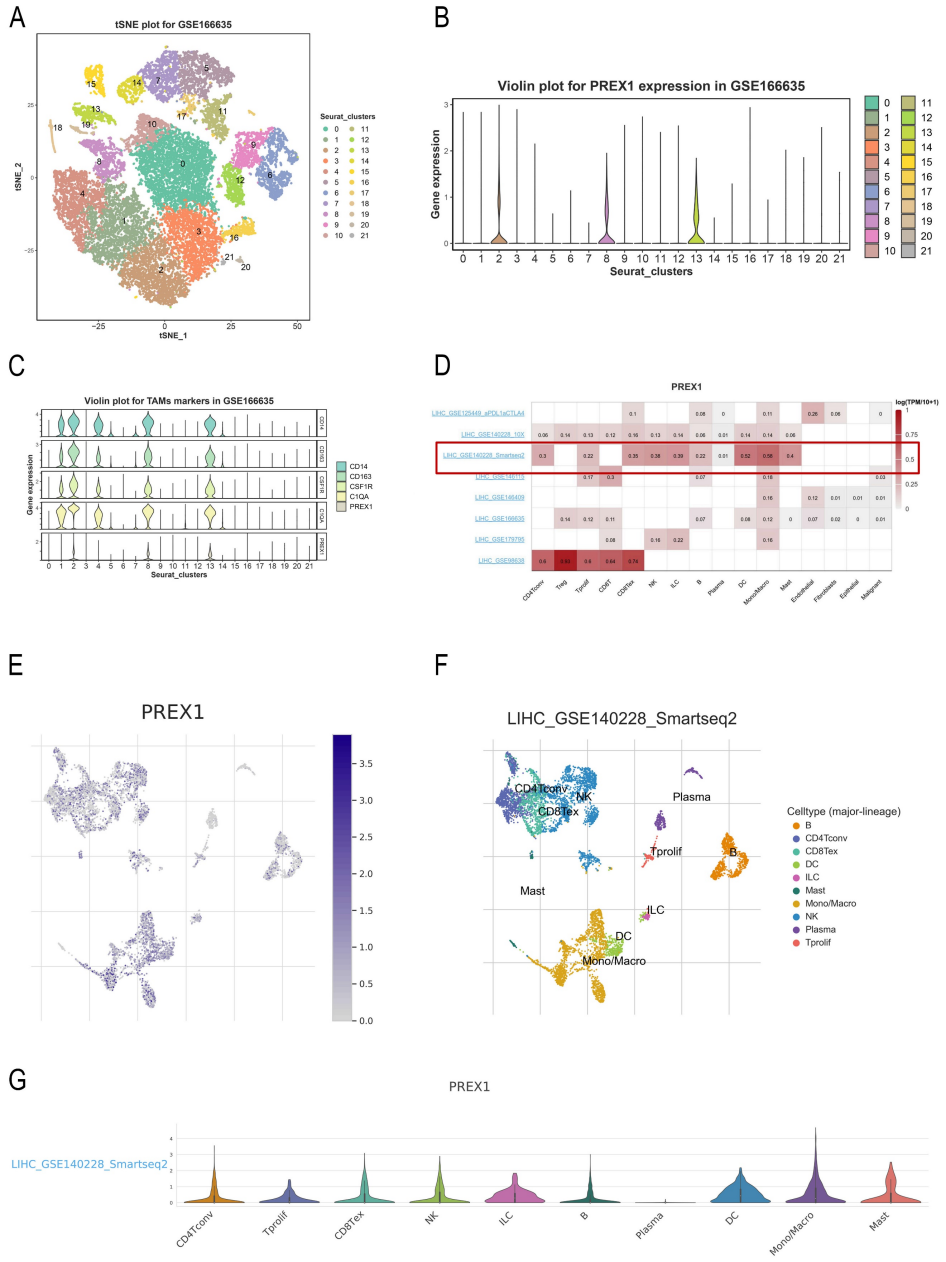
PREX1 expression and cells in LIHC were evaluated by single cell analysis. (A, B) PREX1 was expressed in the highest abundance in cluster 2,8,13 by the analysis of single cell dataset (GSE166635). (C) Single cell analysis of GSE166635 dataset found significant correlation between PREX1 and macrophage-related marker genes such as CD163, CSF1R, CD14 and C1QA. (D) Eight single-cell datasets showed expression of PREX1 in LIHC from TISCH; (E-G) The main distributions of PREX1 on cell types in the LIHC _GSE140228_Smartseq2.

For exploring the relationship between PREX1 expression and cells, we applied a single-cell RNA sequencing dataset of LIHC (GSE166635) to conduct bioinformatics analysis. The sample included two cases of liver tumors and two cases of normal liver tissues. After principle component analysis (PCA), we acquired 22 cell clusters and showed them in the t-SNE plot (Figure 7A).

The bar chart suggests that the highest expression of PREX1 were mainly found in cluster 2,8,13 (Figure 7B). Then, we detected that PREX1 was co-expressed with many macrophage markers, such as CD163, CSF1R, CD14 and C1QA (Figure 7C; Supplementary Figure 4). Bulk sequencing also indicated that PREX1 was correlated macrophages markers (Supplementary Figure 5, 6). To further validate the distribution of PREX1 expression in LIHC, 8 related single-cell datasets from TISCH was analyzed. Our study showed that PREX1 was broadly expressed in various immune clusters (Figure 7D). In the LIHC _GSE140228_Smartseq2 dataset, 7074 cells from 6 patients with LIHC were analyzed; the highest expression of PREX1 were mainly found in macrophages (Figure 7E-G).

### Relationship between PREX1 expression and sensitivity to immunotherapy in LIHC

**Figure 8:**
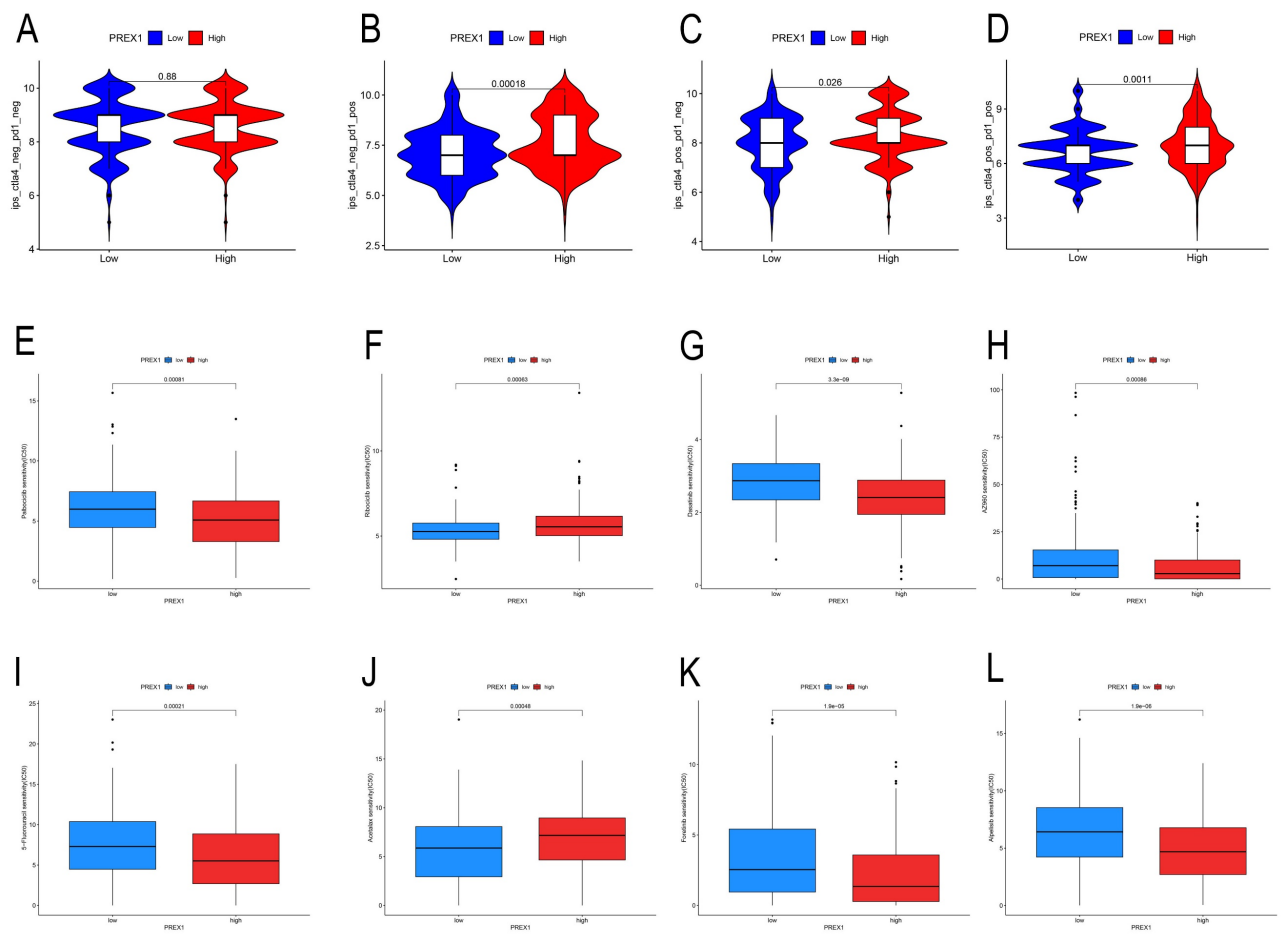
PREX1 is significantly associated with immunotherapy sensitivity in LIHC. (A-D) The sensitivity of immune checkpoint inhibitors (PD1 and CTLA4 inhibitors) in the high and low PREX1 expression groups. PREX1 was associated with the efficacy of chemotherapy medicines. (E-L) Comparison of TCGA-LIHC groups with high and low PREX1 expression in terms of chemotherapeutic effects by R package.

Our findings displayed that high PREX1 level group were more likely to benefit from immunosuppression therapy. There were no significant differences in the CTLA4-PD1-group (Figure 8A). In the CTLA4-PD1+, CTLA4+PD1-group and CTLA4+PD1+ groups, the higher the PREX1 expression, the better the effect of receiving immunotherapy (Figure 8B-D). These results suggest that LIHC patients with high PREX1 expression may exhibit better responses to immunosuppression treatment.

### Relationship between PREX1 expression and drug sensitivity in LIHC

In this study, we found that PREX1 played a vital role in tumor progression in LIHC. Thus, we seek chemo drugs to thwart the PREX1-modulated oncogenic process. In this study, we analyzed some drugs in both high- and low-PREX1 expression groups, including Dasatinib, Ribociclib, Alpelisib, Foretinib, Palbociclib and 5-Fluorouracil, with remarkable discrepancy in their IC50 values (Figure 8E-L).

### Depletion of PREX1 significantly inhibited the proliferation and invasion in LIHC cells

**Figure 9:**
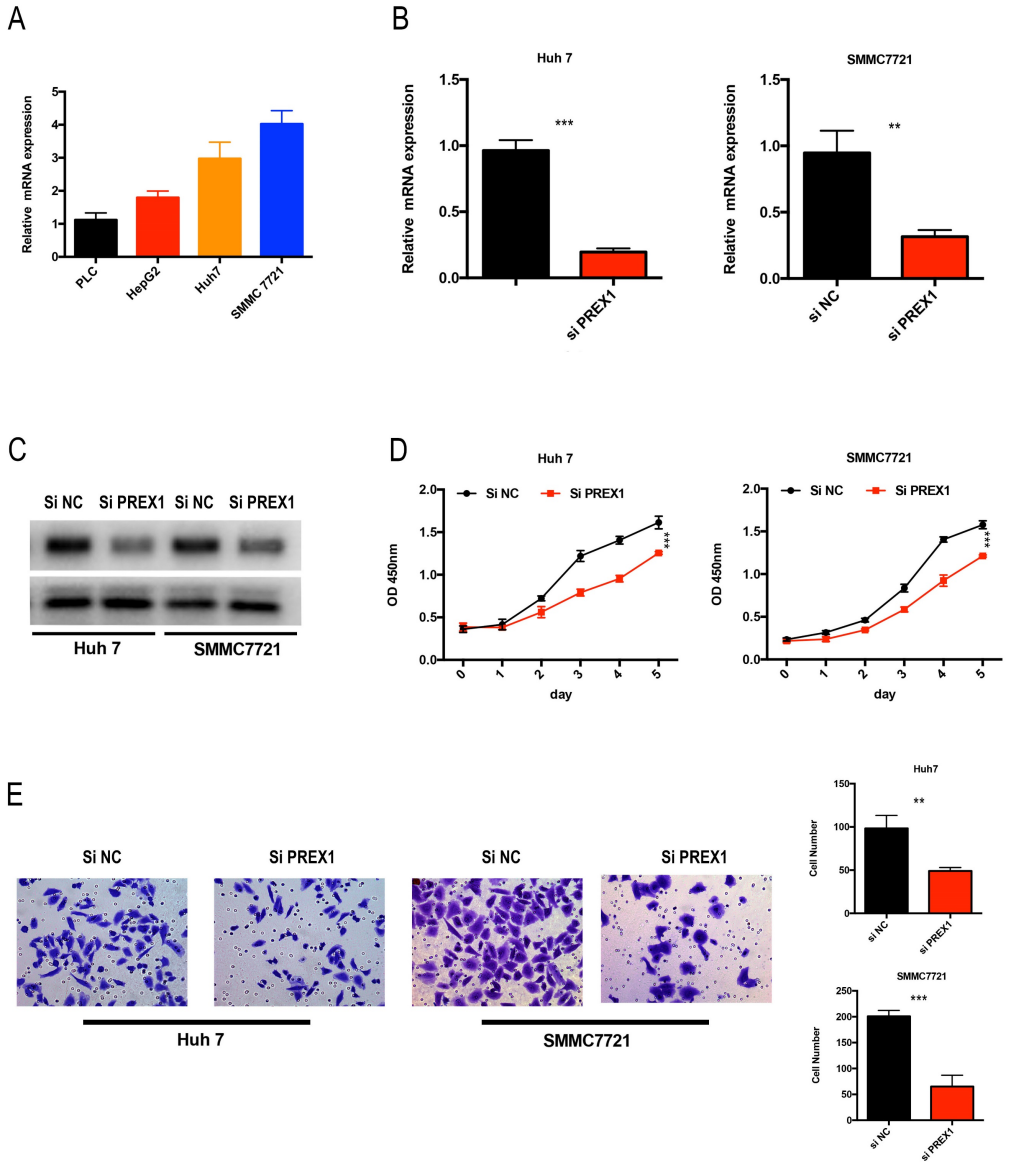
Downregulation of PREX1 repressed the proliferation and invasion ability of LIHC cells. (A) PREX1 expression was higher in Huh7 and SMMC7721 cells than that in other LIHC cell lines by qPCR assay. (B-C) The efficacy of PREX1-siRNA was certified by qPCR and Western Blot in Huh7 and SMMC7721 cells. (D) The proliferation ability of LIHC cells with PREX1 downregulation verified by CCK8 assay. (E) The invasion ability of LIHC cells with PREX1 downregulation confirmed by transwell assay.

As PREX1 played a critical role in LIHC tumor progression, we analyzed the expression of PREX1 in LIHC cell lines. Huh7 and SMMC7721 cells were chose to transfect with si-NC or si-PREX1, of whose expression was relative upregulated (Figure 9A). Next, the efficacy of siRNA-PREX1 was examined by qPCR and Western Blot (Figure 9B, C). Then, CCK8 and transwell assays were employed to measure proliferative and invasive abilities of LIHC cells. The results showed that depletion of PREX1 can significantly suppress proliferation and invasion in LIHC (Figure 9D-E).

## Discussion

LIHC remains a threatening disorder globally and causes a huge public burden. Despite surgical resection may provide clinical benefits for early patients, the prognosis of patients with advanced stage is still frustrating. As a results, it is extremely important to screen prospective biomarkers for LIHC diagnosis and treatment options. Previous research suggested that depletion of PREX1 could hinder the recruitment of monocyte, macrophages and neutrophils 14, 15, 29. Thus, we suppose that PREX1 may play an immune-related role in LIHC which needs further experiments to validate.

To conduct a deeper study of PREX1, we analyzed the co-expressed genes of PREX1 in LIHC by using R package. Our results revealed that PREX1 was positively correlated with KCTD12, HEG1, RASSF2, FMNL3 and RCSD1, with the most significant correlation with RCSD1. Previous research has demonstrated that RCSD1 is positively related to infiltrating macrophages in lung cancer 30. FMNL3 play a critical role in the recruitment of TILs and inflammatory tumor microenvironment 31. Functional analysis uncovered that PREX1 was associated with PI3K-Akt signaling pathway; this result is consistent with previous studies 32. Interestingly, PREX1 expression was also mainly enriched in immune-related pathways, implying a complex role of PREX1 in biological process of LIHC.

The species and proportions of TILs in the immune microenvironment may be associated with tumor development 28. For example, macrophages are versatile cells with functions such as maintaining tissue development and homeostasis, clearing cell waste, clearing pathogens, and producing cytokines. Although macrophages serving immunomodulatory function in LIHC microenvironment, but its influence on immune cells is often destroyed by tumor cells, contributing to immune tolerance and invasion. Targeting macrophages is a potential strategy for cancer treatment, which include: preventing their recruitment, reversing macrophages to M1 type and targeting immunosuppressive molecules produced by macrophages. Our previous studies disclosed that targeting disrupted tumor-associated macrophage could help prevent tumor immune escape and improve the efficacy of PD-L1 treatment 33. In this study, the enrichment of PREX1 in immune cells, especially in macrophages has been identified by CIBERSORT algorithm and single cell analysis. Interestingly, in single cell analysis, there may be a positive correlation between PREX1 and M2 macrophages, which is inconsistent with the results from CIBERSORT that showed a positive correlation between PREX1 and M1 macrophages. Although M1-M2 paradigm has become dominant in macrophage biology, it is an oversimplification that fails to describe the multitude of macrophage states within tumors. Emerging evidences highlighted the diversity and heterogeneity of macrophages in cancer 34. For example, single cell transcriptomics indicated that tumor-associated macrophages (TAMs) co-express M1 and M2 markers 35. In 2022, Ma *et al.* identified seven distinct subsets of TAMs that were consistently present across various types of cancer 36. So, in our work, classical M1-M2 paradigm is not enough to explain the association between PREX1 and macrophage. This result needs more experiment to validate in the future, such as spatial transcriptome.

Furthermore, this study found a significant correlation between PREX1 expression and key immune modulators such as MHC molecules, immune inhibitors, immune stimulators, chemokines and its receptor. Another study has shown that PREX1 can be regulated by CXCR4, which in turn affects the secretion of IL-2 and IL-10 in T cells 37. Considering the complexity of immune regulation, PREX1 may be involved in various signaling pathways and regulatory networks that are decisive for tumor development. These findings hold promise for shedding light on the regulatory mechanisms of PREX1 within the immune microenvironment of LIHC and may conduce to the novel therapeutic strategies targeting PREX1.

Monoclonal antibodies targeting immune checkpoint molecules have shown clinical benefits in various cancer treatments 38. However, the response rate to immune checkpoint treatment is only 15% -20% in LIHC, far lower than other solid tumors 39, 40. Therefore, seeking prospective biomarkers that predict the response to immunotherapy in liver cancer is an urgent task. Several biomarkers have been applied to predict efficacy of immunosuppressive therapies for LIHC patients, the results are still discouraging. Our findings pointed out that patients with high PREX1 level had a higher IPS score than those with low PREX1 level, and PREX1 may serve as a prospective indicator for immunotherapy in LIHC.

To further explore the function of PREX1 in LIHC, siRNA was applied to decrease the expression of PREX1. The results showed that downregulation of PREX1 could restrain the ability of proliferation and migration in LIHC. Thus, PREX1 is an important tumor promoter in LIHC development *in vitro*.

This study informed that PREX1 may have a fundamental role in tumor immunity of LIHC, but limitations still remain. More experiments are needed to support our results about the role of PREX1 in LIHC owing to the complexity of molecular biological mechanisms in tumorigenesis.

## Conclusion

Taken together, this study clearly states that upregulated PREX1 exhibited a remarkable association with dismal prognosis in LIHC. Through multiple databases, we found that PREX1 played an important role in shaping the immune microenvironment of LIHC. Intriguingly, we also detected that PREX1 was closely linked with sensitivity to immune checkpoint inhibitors and chemotherapy drugs. Overall, we have completed a comprehensive evaluation of PREX1, uncovering that it plays a critical role in tumor immune environment and serves as an indicator for dismal prognosis in LIHC patients.

## Supplementary Material

Supplementary figures and tables.

## Abbreviations

LIHC: Liver hepatocellular carcinoma
PREX1: PtdIns (3,4,5)P3-dependent Rac exchanger 1
TCGA: The Cancer Genome Atlas
OS: Overall survival
DFS: Disease-free survival
TIMER: The Tumor Immune Estimation Resource
UALCAN: University of Alabama Cancer Database
DEGs: Differentially expressed genes
GO: Gene Ontology
KEGG: Kyoto Encyclopedia of Genes and Genomes
GSEA: Gene Set Enrichment Analysis
PCA: Principal component analysis
IPS: Immunophenoscores
TCIA: The Cancer Immunome Database
FBS: Fetal bovine serum
DMEM: Dulbecco's modified Eagle's medium
PBS: phosphate buffer saline
TILs: Tumor infiltrating lymphocytes
