# Supplementary material for: Integrated Single-cell and Transcriptome Sequencing Analyses Identified PREX1 as an Immune-related Prognostic Biomarker for Liver Hepatocellular Carcinoma
Source: Int J Med Sci. 2024 Jun 3;21(8):1559–74. doi: 10.7150/ijms.94812 (PMC11186429; doi:10.7150/ijms.94812)
Supplement: Supplementary file 1 — Supplementary figures and tables. [file ijmsv21p1559s1.pdf]

Supplementary Material

Integrated Single-cell and Transcriptome Sequencing Analyses Identified PREX1 as an Immune-related Prognostic Biomarker for Liver Hepatocellular Carcinoma

Supplementary Table 1. Gene description of GO enrichment analyses of PREX1.

Supplementary Figure 1. Clinical information for two different expression groups of PREX1.

Supplementary Figure 2. The correlation between genes significantly related to PREX1 (KCTD12, HEG1, RASSF2, FMNL3 and RCSD1).

Supplementary Figure 3. The differences of PREX1 expression in TME scores by the ESTIMATH algorithm and the Wilcoxon test.

Supplementary Figure 4. Analysis of single-cell data found significant associations between PREX1 and macrophage-associated marker genes.

Supplementary Figure 5. The correlation between PREX1 and macrophage-associated marker genes by analysis of GEPIA.

Supplementary Figure 6. The correlation between PREX1 and macrophage-associated marker genes by analysis of TCGA-LIHC samples.

Supplementary Table 1. Gene description of GO enrichment analyses of PREX1.

ID	Description	GeneRatio	p-value
GO:0003823	antigen binding	108/1448	7.66E-75
GO:0034987	immunoglobulin receptor binding	56/1448	1.35E-43
GO:0005201	extracellular matrix structural constituent	64/1448	1.72E-27
GO:0140375	immune receptor activity	46/1448	1.43E-16
GO:0030020	extracellular matrix structural constituent conferring tensile strength	20/1448	3.91E-12
GO:0005539	glycosaminoglycan binding	51/1448	1.71E-11
GO:0002696	positive regulation of leukocyte activation	144/1546	4.86E-53
GO:0050867	positive regulation of cell activation	146/1546	1.88E-52
GO:0051251	positive regulation of lymphocyte activation	132/1546	7.02E-51
GO:0050853	B cell receptor signaling pathway	75/1546	4.15E-47
GO:0002768	immune response-regulating cell surface receptor signaling pathway	119/1546	6.44E-47
GO:0002443	leukocyte mediated immunity	142/1546	1.42E-46
GO:0019814	immunoglobulin complex	109/1589	1.73E-77
GO:0042101	T cell receptor complex	91/1589	6.77E-63
GO:0009897	external side of plasma membrane	158/1589	5.13E-60
GO:0098802	plasma membrane signaling receptor complex	123/1589	1.52E-53
GO:0098802	plasma membrane signaling receptor complex	123/1589	1.52E-53
GO:0042571	immunoglobulin complex, circulating	55/1589	7.21E-43
GO:0062023	collagen-containing extracellular matrix	96/1589	2.16E-20

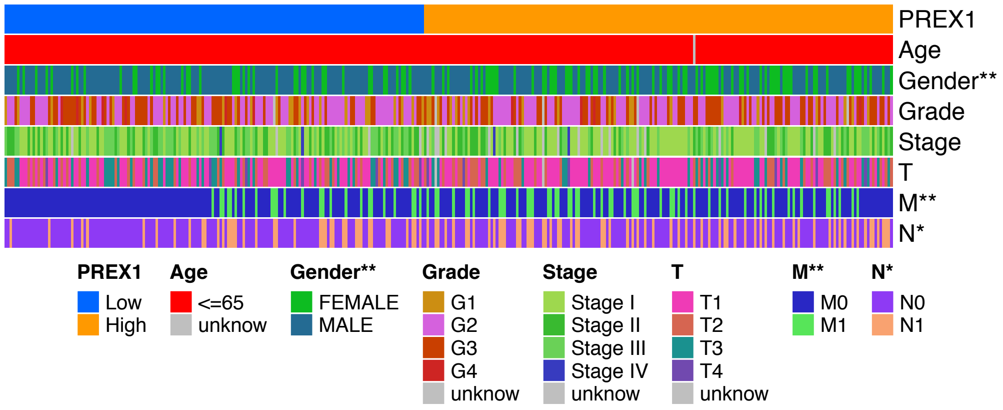

Supplementary Figure 1. Clinical information for two different expression groups of PREX1.

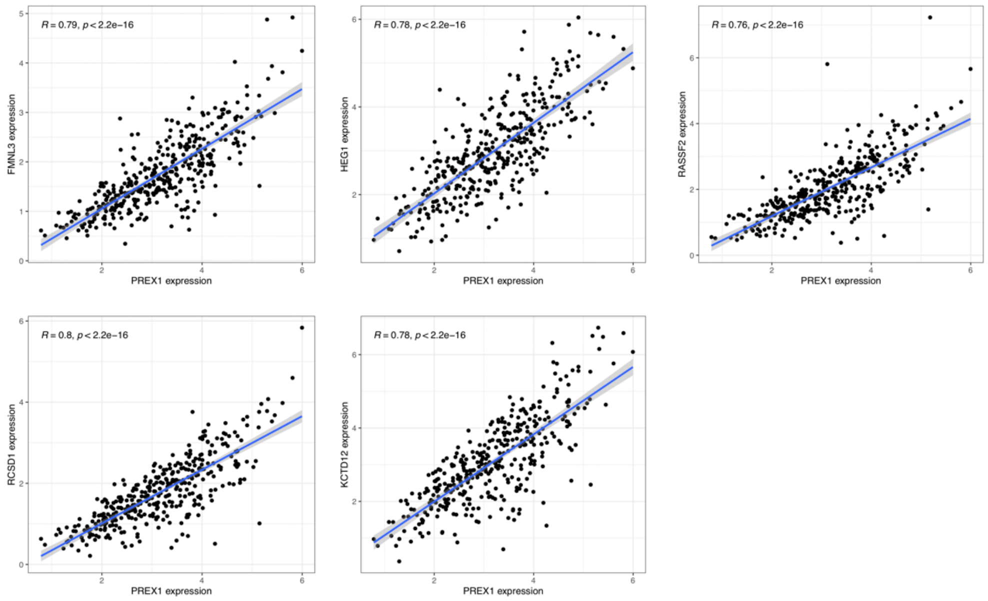

Supplementary Figure 2. The correlation between genes significantly related to PREX1 (KCTD12, HEG1, RASSF2, FMNL3 and RCSD1).

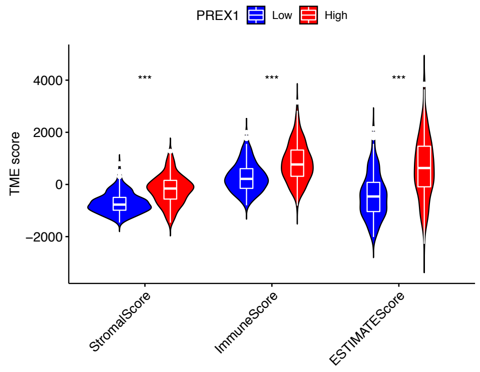

Supplementary Figure 3. The differences of PREX1 expression in TME scores by the ESTIMATH algorithm and the Wilcoxon test.

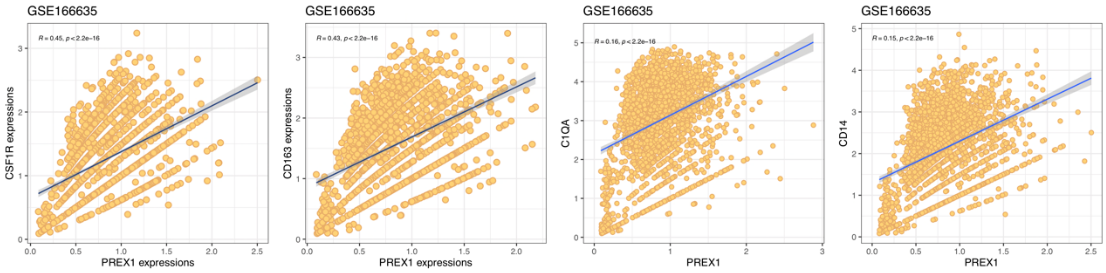

Supplementary Figure 4. Analysis of single-cell data found significant associations between PREX1 and macrophage-associated marker genes.

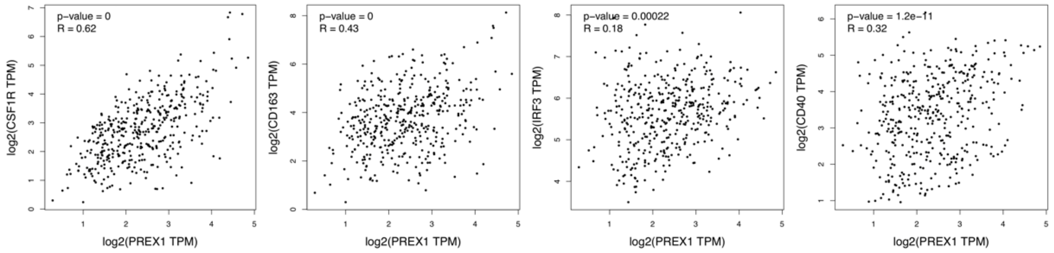

Supplementary Figure 5. The correlation between PREX1 and macrophage-associated marker genes by analysis of GEPIA.

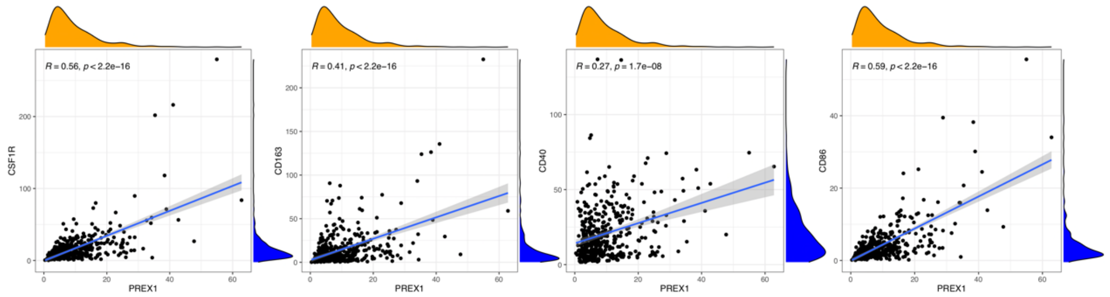

Supplementary Figure 6. The correlation between PREX1 and macrophage-associated marker genes by analysis of TCGA-LIHC samples.
